# The Current and Future Landscape of Structural Heart Interventions

**DOI:** 10.14797/mdcvj.1251

**Published:** 2023-05-16

**Authors:** Sachin S. Goel

**Affiliations:** 1 Houston Methodist DeBakey Heart & Vascular Center, Houston Methodist, Houston, Texas, US

**Keywords:** transcatheter aortic valve replacement (TAVR), aortic stenosis, surgical aortic valve replacement, mitral transcatheter edge-to-edge repair, transcatheter mitral valve replacement

Dr. Alain Cribier performed the first-in-human transcatheter aortic valve replacement (TAVR) procedure in April 2002 in Rouen, France. Over the next two decades, unique collaboration ensued between cardiologists, cardiac surgeons, and the medical device industry. This led to a series of very high-quality and well-designed, well-performed randomized controlled trials, which have shown that TAVR is safe and effective in treating patients with severe symptomatic aortic stenosis across all surgical risks. This led to a monumental shift in the treatment of patients with aortic stenosis: in the United States, the annual numbers of TAVR now exceed those of surgical aortic valve replacement. At the 71st Annual Scientific Sessions of the American College of Cardiology held in April 2022, we celebrated the 20th anniversary of Dr. Cribier’s first human TAVR procedure. Growing experience, coupled with continued innovation, have consistently improved the safety and outcomes of TAVR.

Concomitantly, we have seen growth in the mitral space with the evolution of mitral transcatheter edge-to-edge repair (TEER) technology, which is now approved for treatment of patients with severe degenerative and functional mitral regurgitation. For patients who are not suitable for TEER, transcatheter mitral valve replacement is emerging as a novel option. The “forgotten” tricuspid valve is no longer forgotten, and ongoing trials are evaluating edge-to-edge repair and transcatheter tricuspid valve replacement options. The last decade also has seen innovations in transcatheter left atrial appendage closure technology for stroke prevention in patients with atrial fibrillation, as well as transcatheter interventions in patients with adult congenital heart disease. Of course, all of these interventions depend heavily on multimodality cardiac imaging, without which we would not see the results that we achieve today.

Given the tremendous growth, expanding indications, and ongoing innovations in the structural heart space, we developed this issue of the *Methodist DeBakey Cardiovascular Journal* with a focus on providing our readers an update on the current landscape of structural heart interventions. We kick off this issue with “What’s New with TAVR?” by Drs. Syed Zaid, Gilbert Tang, and colleagues. This beautifully written and illustrated review discusses the latest advances in transcatheter delivery systems and devices for TAVR, including the latest iterations of the balloon expandable and self-expandable transcatheter heart valve systems, namely the Sapien X4 valve and the Evolut FX systems, the 3rd generation Navitor transcatheter heart valve (THV), and the Acurate Neo2 THV. JenaValve represents an exciting new technology designed for transcatheter treatment of pure native aortic regurgitation and fulfilling this unmet need. The authors also review the potential role of Resilia tissue with anticalcification properties, Anteris DurAVR single piece 3D leaflet technology, and Foldax Tria—all with the potential of extending durability profiles for next-generation transcatheter heart valves.

Next, Drs. Parth Desai, Michael Reardon, and coauthors focus on TAVR long-term outcomes and durability. A common question asked by patients being evaluated for TAVR in the valve clinic is “Doc, how long is this valve going to last?” This is becoming increasingly important given expanding utilization of TAVR in younger patients with longer life expectancy. In this review, the authors discuss the mid- to long-term (> 5 years) clinical outcomes from the landmark TAVR trials and analyze the available long-term durability data. The importance of using standardized definitions of bioprosthetic valve dysfunction is emphasized.

Switching gears, Drs. Habib Layoun, Samir Kapadia, and colleagues from The Cleveland Clinic discuss an important topic: patient selection for mitral transcatheter edge-to-edge repair. The authors discuss relevant published data on the procedure in primary and secondary mitral regurgitation and emphasize the importance of advanced 2D and 3D imaging for patient selection and intraprocedural guidance to optimize procedural outcomes.

From there, Drs Hiroki Ueyama, Adam Greenbaum, and coauthors from Emory University Hospital present a state-of-the-art review on transcatheter mitral valve replacement using the Sapien platform in failed bioprosthetic valves (valve-in-valve), surgical annuloplasty rings (valve-in-ring) and native valves with mitral annular calcification (valve-in-MAC). Their review summarizes the indications, contemporary trends in utilization, unique challenges, pre- and periprocedural planning, and clinical outcomes with the US Food and Drug Administration (FDA)-approved valves used in transcatheter mitral valve replacement.

The third and final transcatheter mitral review, by Drs. Joe Aoun, Michael Reardon, and myself, focuses on TMVR with dedicated devices, none of which are FDA approved in the US, although several are currently being evaluated in clinical trials. We briefly discuss several devices that are promising with emerging data—Tendyne, Intrepid, AltaValve, Sapien M3, Cephea, and Highlife, to name a few. Success in this field will require excellence in cardiac imaging, patient selection, and continued innovations in valve anchoring, decreases in delivery sheath profiles, and better understanding and solutions to decrease the risk of left ventricular outflow tract obstruction, which appears to be the Achilles heel of TMVR technology.

Next, Drs. Colin Barker and Kashish Goel from Vanderbilt University Medical Center focus on transcatheter tricuspid valve interventions. They summarize the anatomy and pathophysiology of tricuspid regurgitation and discuss patient evaluation, limitation of surgery for isolated tricuspid regurgitation, and the recently presented and published TRILUMINATE trial, reporting the safety and effectiveness of transcatheter tricuspid valve repair with the TriClip. They also explore the ongoing trial of the Pascal device for tricuspid TEER, and they offer a glimpse into the future with several orthotopic and heterotopic transcatheter tricuspid valve replacement strategies that are being studied, with several novel therapies on the horizon.

After these highly informative and contemporary reviews on transcatheter valve interventions, Drs. Gordon Wong and Gagan Singh from the University of California Davis Medical Center discuss the indications for transcatheter left atrial appendage closure and the evidence evaluating the use of various device therapies currently available, including the Watchman and Amulet devices as well as those in development. They also discuss the role of intraprocedural guidance with intracardiac echocardiography as an alternative to conventional transesophageal echocardiographic guidance and the nuances surrounding various post-implantation antithrombotic regimens.

Drs. Rody Bou Chaaya, Chun Huie Lin, and coauthors from Houston Methodist provide an excellent illustrated review on “Percutaneous Structural Interventions in Adult Congenital Heart Disease.” These range from closure of patent ductus arteriosus, atrial and ventricular septal defects, aortopulmonary and venovenous collaterals in patients with prior Fontan procedure, stenting aortic coarctation, angioplasty and stenting of the right-ventricular-to-pulmonary artery conduit, pulmonary artery, pulmonary veins, Fontan conduits, and atrial switch baffle stenoses to transcatheter pulmonary valve replacement and transcatheter tricuspid valve interventions. They emphasize the need for continuous innovation for patients with adult congenital heart disease to increase collaboration with industry and regulatory bodies to develop dedicated devices.

Last but not least, Drs. Amr Telmesani, Dipan Shah, and colleagues provide a superb overview on the “Role of Multimodality Imaging in Transcatheter Structural Interventions.” They discuss the role of echocardiography in pre- and intraprocedural guidance for TEER, cardiac computed tomography for procedural planning of TAVR, transcatheter mitral valve replacement and assessing important issues with THVs, such as hypoattenuated leaflet thickening, and the strengths of cardiac magnetic resonance imaging in accurate volumetric assessment of valvular regurgitation and chamber size quantification.

Finally, Dr. Devang Parikh presents important pearls in the evaluation of an elderly patient being considered for TAVR by way of a brief case vignette, and he stresses the importance of a multidisciplinary heart team approach, appropriate risk stratification based on published risk prediction tools in addition to anatomy, clinical status and patient frailty, and the role of imaging in preprocedural planning for optimal patient outcomes.

We are most grateful to our experts, who have provided up-to-date reviews on key topics that are crucial to the current landscape of structural heart interventions. We hope that the readers will find these interesting, stimulating, and applicable to their clinical practice.

## Guest Editor Biography

The editors of the *Methodist DeBakey Cardiovascular Journal* express our appreciation to Dr. Sachin S. Goel for his guidance and insight in curating this issue on structural heart interventions.

## Sachin S. Goel, MD

**Figure:**
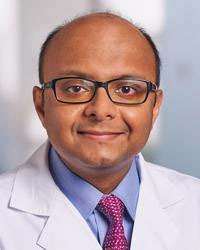


Dr. Sachin Goel is an interventional cardiologist at the Houston Methodist DeBakey Heart & Vascular Center, specializing in structural heart interventions and percutaneous coronary interventions. He serves as the medical director for structural heart interventions at the same institution and performs transcatheter aortic valve replacement, transcatheter edge-to-edge repair (MitraClip), transcatheter mitral valve replacement, catheter based tricuspid valve interventions, balloon aortic and mitral valvuloplasty, transcatheter paravalvular leak closure, atrial septal defect and patent foramen ovale closure, and transcatheter left atrial appendage occlusion.

Dr. Goel’s research focuses on outcomes of patients undergoing transcatheter valve interventions. He is an investigator for several ongoing multicenter trials evaluating safety and efficacy of novel catheter-based valve interventions. Dr Goel is a fellow of the American College of Cardiology and the Society for Cardiovascular Angiography and Interventions. He also serves as a member of The Society for Cardiovascular Angiography and Interventions (SCAI)’s Structural Heart Disease Council.

